# Racial-ethnic differences in impulsivity and compulsivity in recreational gambling

**DOI:** 10.1016/j.comppsych.2019.152153

**Published:** 2020-02

**Authors:** Samuel R. Chamberlain, Jon E. Grant

**Affiliations:** a Department of Psychiatry, University of Cambridge, UK; b Cambridge and Peterborough NHS Foundation Trust, UK; c Department of Psychiatry & Behavioral Neuroscience, University of Chicago, United States of America

**Keywords:** Addiction, Cognition, Gambling, Impulsivity, Phenomenology, Race

## Abstract

**Background:**

Prior data indicate high rates of problematic gambling in some racial-ethnic minority groups, yet research into mechanisms contributing to these associations is scant.

The aim of the present study was to examine whether impulsivity and compulsivity differ across racial-ethnic groups in recreational gamblers.

**Methods:**

Young adult non-treatment seeking recreational gamblers were recruited from the general community. Presence of mental health diagnoses (including gambling disorder) was exclusionary. Participants completed clinical interviews, questionnaires, and cognitive tasks germane to impulsivity and compulsivity.

**Results:**

202 recreational gamblers (63.5% males) had mean (standard deviation) age 23.8 (2.7) years and identified using the following racial-ethnic identities: Caucasian (*N* = 145), African-American (*N* = 41), and Asian (*N* = 16). Groups did not differ on age, gender, education, or impulsivity measures. Compared to the Caucasian group, the African-American group reported significantly higher endorsement of sub-syndromal disordered gambling, higher compulsivity scores, and exhibited decision-making decrements on the Gambling Task. The Asian and Caucasian groups did not differ on any measure.

**Conclusions:**

This study suggests that young adult African-American recreational gamblers may experience greater levels of subsyndromal gambling compared to other racial-ethnic groups, and this appears linked with aspects of compulsivity. Future work should evaluate gambling longitudinally to better understand nuanced presentations across different groups, including in other age groups.

## Highlights

• This is the first study to assess differences in impulsivity and compulsivity as a function of racial-ethnic group in recreational gamblers.
• African-Americans endorsed significantly more subsyndromal symptoms of gambling disorder compared to White Caucasians
• We found no significant differences between African-Americans, White Caucasians, and Asians on measures of impulsivity, but marked differences were found between racial-ethnic groups for measures of compulsivity.

## 1 Introduction

Gambling is a commonplace activity across cultures, and while many people gamble without untoward consequences, a subset of people develop gambling disorder, a mental health disorder characterized by persistent, recurrent maladaptive patterns of gambling behavior and functional impairment [1]. National initiatives have highlighted the importance of understanding racial-ethnic differences in the mental health context (http://minorityhealth.hhs.gov). High rates of problematic gambling have been reported in racial-ethnic minority groups, yet there is a severe poverty of research into the underlying factors that might account for these associations [2]. Studies have also indicated that higher rates of gambling in certain racial-ethnic minority groups may be due to different cultural norms, acculturation, and attitudes towards gambling [[3], [4], [5]], as well as lower rates of social inclusivity [6]. Additionally, psychological research has generally failed to focus enough on differences in racial-ethnic groups more widely for a variety of reasons; yet such research has many potential benefits for wider society (for detailed discussion see [7]).

In one study, higher rates of gambling disorder were found in black as opposed to white individuals, but racial and ethnic groups generally had similar symptom patterns, time courses, and rates of treatment seeking [3]. Some studies suggest that clinical differences may exist in the presentation of gambling (and disordered gambling) between different racial-ethnic groups. For example, one study in gamblers found that African-American individuals were more likely to report hypomania, or any substance use disorder, as well as mood disorder [8]. Among Asian-American adolescents, stronger associations were observed between at-risk/problem gambling and smoking cigarettes (interaction odds ratio = 12.6) than in Caucasian adolescents [4]. In a study of people calling into a gambling helpline, African-American individuals were more likely than Caucasian individuals to report longer duration of gambling problems, but were less likely to report having received mental health treatment [9]. A large study of university students (*n* = 3058) found that Asian participants gambled less frequently than Caucasians or Hispanic/Latino(a)s, but spent more money overall on gambling than participants who were African-American (AA)/Black or Hispanic/Latino(a) [10].

In addition to demographic and clinical parameters potentially being relevant to understanding racial-ethnic differences in mental health disorders such as gambling disorder [11], and the above-mentioned research implicating issues such as social norms, acculturation, and inclusivity [[3], [4], [5], [6]], the concepts of impulsivity and compulsivity may also be relevant. Impulsivity is a tendency towards behaviors that are risky and unduly hasty, leading to untoward outcomes [12]. Compulsivity is a tendency towards behaviors that are repetitive and difficult to suppress, leading to untoward outcomes [1]. The RDoC initiative highlights the need to explore relevant dimensions of symptoms and relevant traits in mental health, rather than only categorical diagnosis [13].

In a recent Delphi study of experts' views on substance and behavioral addictions, the concepts of impulsivity and compulsivity were both felt to be important [14]. In one study of 315 problem gamblers who completed a delay-discounting questionnaire involving choices between a smaller amount of money delivered immediately and a larger amount delivered later, results showed that white gamblers discounted delayed money at lower rates than African Americans and Hispanics, even after controlling for confounding variables [15]. In a prior study in people with gambling disorder, black individuals reported more symptoms of disordered gambling, and higher self-report obsessive-compulsive traits (Padua Inventory), than white individuals [16]. Additionally, gambling disorder was associated with greater deficits on attentional set-shifting (i.e. task can be seen as related to compulsivity since inability to inhibit attentional bias and show flexible responding leads to errors on the task) and gambling task performance in black compared to white individuals with gambling disorder. Deficits on decision-making tasks are associated with gambling disorder, according to meta-analysis [17]. Racial-ethnic associations with cognitive function in gambling are under-studied, but may shed lights of neurobiological pathways.

Therefore, the aim of the current study was to explore differences in impulsivity and compulsivity between racial-ethnic groups of recreational gamblers. We quantified impulsivity and compulsivity using questionnaires and neurocognitive tasks. Since mental health disorders are themselves associated with elevated scores on such instruments, we included only treatment non-seeking recreational gamblers who did not have any mental health diagnoses according to gold-standard clinical interviews. We hypothesized that there may be higher levels of impulsivity and compulsivity in African-American as compared to Caucasian recreational gamblers, based on previous literature, and the finding that particular racial-ethnic status appears to be linked with higher risk of disordered gambling.

## 2 Methods

### 2.1 Subjects

Non-treatment seeking participants aged 19–29 years were recruited using media advertisements in two large US cities. Inclusion criteria were having gambled at least five times in the preceding year, the provision of informed consent, and an ability to understand the study procedures. Mental health disorders (such as mood and anxiety disorders, substance use disorders, gambling disorder, and impulse control disorders) were exclusionary (for screening methods see later).

The study procedures were carried out in accordance with the Declaration of Helsinki. The Institutional Review Board of the University of Chicago approved the study and the consent statement. After all study procedures were explained, subjects provided voluntary written informed consent. Participants were compensated with a $50 gift card for a local department store.

### 2.2 Clinical and questionnaire assessments

Demographic information was also collected from participants as follows: age, gender, racial-ethnic group (participants self-identified their racial group based on a single open-ended question), education level, consumption of alcohol, and consumption of nicotine. Additionally, participants were asked if they had any first-degree family members with a history of an addictive disorder. Body mass index (BMI) was also measured, since elevated BMI has previously been reported in gambling disorder.

Clinical interviews were undertaken by trained raters using the Mini-International Neuropsychiatric Interview [18], the modified Structured Clinical Interview for Gambling Disorder (SCI-GD) [19], and the Minnesota Impulse Disorders Inventory (MIDI) (Chamberlain and Grant, 2018; [20]). The MINI is a structured clinical interview that assesses whether diagnostic criteria are met for a variety of mainstream mental disorders, such as mood disorders, anxiety disorders, psychosis, and post-traumatic stress disorder. The MIDI is a structured clinical interview designed to assess whether diagnostic criteria are met for impulsive disorders such as hair pulling disorder, kleptomania (compulsive stealing), and binge-eating disorder. The SCI-GD quantifies the number of criteria met for Gambling Disorder, from zero to nine (and can also be used for diagnosis).

Participants completed the following rating scales in order to quantify impulsivity and compulsivity: the Barratt Impulsivity Scale (BIS-11) [21,22], the World Health Organization (WHO) Attention-Deficit Hyperactivity Disorder Scale (ASRS, Part A) [23,24], the Padua Obsessive-Compulsive Inventory (Washington State Revision) [25,26], and the Cambridge-Chicago Trait Compulsivity Scale (CHI-T) [32]. The BIS-11 is a widely used self-report scale quantifying impulsive tendencies. The ASRS is a rating instrument developed by the WHO, designed to screen for the core symptoms of ADHD; it can be used to generate a total score, or as a means of identifying probable ADHD cases. The Padua inventory is a self-report questionnaire designed to capture core symptoms of OCD across a variety of domains, in patient and general population settings. The CHI-T is a short instrument recently developed and validated, which is designed to quantify compulsive tendencies trans-diagnostically.

For more detailed descriptions of the above instruments (including psychometric properties), please see the previous cited validation studies.

### 2.3 Cognitive assessments

We utilized tests selected from the Cambridge Neuropsychological Test Automated Battery (CANTAB). The cognitive domains of interest were response inhibition, set-shifting, and decision-making. We focused on these areas after considering the existing literature on gambling disorder and at-risk gambling [17].

We assessed response inhibition using the Stop-Signal Task [27,28]. Subjects viewed a series of directional arrows appearing one per time on-screen, and made speeded motor responses depending on the direction of each arrow (left button for a left-facing arrow, and vice versa). On a subset of trials, an auditory stop-signal occurred (‘beep’) to indicate to volunteers that response suppression was needed for the given trial. This task uses a dynamic tracking algorithm to calculate the ‘stop-signal reaction time’, which is an estimate of the time taken by the given volunteer's brain to suppress a response that would normally be undertaken.

Set-shifting was measured using the Intra-Dimensional/Extra-Dimensional Set-Shift task (IED) [29]. This task is analogous to the Wisconsin Card Sorting task, but is capable of decomposing more aspects of flexible learning. During the task, participants had to learn rules about which of two presented pictures was “correct” based on feedback provided on-screen. By examining the ability of individuals to learn rules through feedback over nine task stages, the task quantified the total number of errors made, adjusted for task stages that were not attempted. This was the measure of interest on the task.

Decision-making was quantified using the Cambridge Gambling Task (CGT) [30]. There were four practice trials followed by eight blocks of nine trials. At the start of each block, the ‘cumulative points’ setting on the task was reset to 100. On each trial, subjects were shown a set of red and blue boxes, totaling ten. The ratio of red:blue boxes were varied over the course of the task pseudo-randomly (box-ratios: 9_1, 8_2, 7_3, 6_4). Subjects were informed that for each trial, the computer had hidden a ‘token’ inside one of the boxes, and that they had to indicate whether they felt the token would be hidden behind a red or a blue box. This choice was made by selecting ‘red’ or ‘blue’ using the touch-screen interface. After making this judgment, subjects were required to gamble a proportion of their points on whether their color choice was correct. The key outcome measures were (i) mean proportion of points gambled; (ii) quality of decision-making (the proportion of trials where the volunteer chose red when red boxes were in the majority and vice versa – i.e. made the logical color choice); and (iii) risk adjustment (tendency to adjust how many points are gambled depending on the degree of risk).

### 2.4 Procedure of data collection

Participants attended an academic assessment center for an in-person visit. The above procedures were conducted in a quiet room. Clinical instruments were conducted by trained personnel; and administration of the neuropsychological tests was also overseen by a trained member of the study team. For self-report questionnaires, the participants completed these in a quiet waiting area with privacy.

### 2.5 Data analysis

Recreational gamblers were grouped according to their self-declared racial-ethic status. Demographic characteristics, impulsive-compulsive scores, and cognitive performance, were compared between the groups using Analysis of Variance (ANOVA), except where indicated in the text (other suitable tests were used for categorical variables). For the group-level tests, statistical significance was defined as *p* < 0.05 Bonferroni corrected for the number of measures in a given category (i.e. p threshold of 0.05/9 for demographic variables, 0.05/6 for impulsive-compulsive scores, and 0.05/5 for cognitive variables). Significant main effects of group were further explored using post-hoc *t*-tests (or suitable non-parametric tests as indicted in the text). JMP Pro software was used for the statistical analyses. *P* values were reported in uncorrected form in the tables for clarity.

## 3 Results

After excluding data for racial-ethnic groups for whom there were insufficient numbers of subjects for analysis (*N* < 15 threshold), the sample comprised *N* = 202 subjects, with mean (standard deviation) age of 23.8 (2.7) years, being 63.5% male. The sample sizes per racial-ethnic group included in the analysis were: White Caucasian (*N* = 145), African-American (*N* = 41), and Asian (*N* = 16).

Table 1 shows the demographic characteristics of the study groups. It can be seen that the groups did not differ in terms of age, gender, education levels, consumption of alcohol, consumption of nicotine, or likelihood of reporting a history of addiction in their first-degree relatives. The African-American group endorsed a significantly higher number of disordered gambling symptoms than the White Caucasian group; whereas the Asian group did not differ significantly from the other groups on this measure. The trend towards differences in BMI between the groups was not significant with Bonferroni correction, and so is not considered further.

**Table 1:** Demographic Characteristics of the Groups. Data refer to mean (SD) or N [%]. Statistical tests are ANOVA, except where indicated “L” for likelihood-ratio chi-square test. * indicates significant ANOVA, Bonferroni-corrected *p* < 0.05. Superscript letters indicate significant pairwise group differences (post-hoc *t*-tests): ^a/b/c^ p < 0.05, ^aa/bb/cc^*p* < 0.01, ^aaa/bbb/ccc^*p* < 0.001.

	White Caucasian (145)^a^	African-American (*N* = 41)^b^	Asian (*N* = 16)^c^	F	P
Age, years	23.6 (2.8)	24.4 (2.6)	23.9 (2.6)	1.143	0.321
Gender, female, N [%]	47 [32.4%]	15 [48.4%]	8 [50.0%]	4.099 L	0.123
Education	3.4 (0.9)	3.1 (0.9)	3.6 (1.0)	1.737	0.179
SCI-PG	0.70 (1.30)^bbb^	2.03 (2.17)^aaa^	1.31 (1.30)	10.905	<0.001*
Age at first gambling, years	14.8 (4.1)	14.1 (3.1)	16.4 (5.3)	1.7261	0.1808
Alcohol consumption, times/week	1.7 (1.4)	1.6 (1.5)	0.9 (1.3)	2.267	0.107
Smoking (nicotine), packs per day equivalent	0.11 (0.27)	0.10 (0.30)	0.05 (0.19)	0.370	0.691
History of addiction in first-degree relative, N [%]	[30.3%]	11 [35.5%]	4 [25.0%]	0.587 L	0.746
BMI, mg/kg2	24.1 (3.9)	27.0 (7.1)	23.3 (6.2)	5.072	0.0072

Table 2 shows the self-reported scores on impulsive and compulsive questionnaires for the study groups. It can be seen that the study groups did not differ from each other in terms of impulsive measures (Barratt Impulsivity Scale, BIS, and the Attention-Deficit Hyperactivity Disorder Scale, ASRS). In comparison to the White Caucasian group, the African-American group reported significantly higher levels of compulsive tendencies (Padua Obsessive-Compulsive Inventory, and the Cambridge-Chicago Trait Compulsivity Scale, CHI-T). The Asian group did not differ from the other groups for Padua Obsessive-Compulsive Inventory scores, but had significantly lower scores than the African-American group on the CHI-T.

**Table 2:** Impulsive and Compulsive scores in the groups. Data refer to mean (SD). Statistical tests are ANOVA. * indicates significant ANOVA, Bonferroni-corrected *p* < 0.05. Superscript letters indicate significant pairwise group differences (post-hoc *t*-tests): ^a/b/c^ p < 0.05, ^aa/bb/cc^*p* < 0.01, ^aaa/bbb/ccc^*p* < 0.001.

	Caucasian (145)^a^	African-American (*N* = 41)^b^	Asian (*N* = 16)^c^	F	P
BIS, attentional impulsivity	15.8 (4.1)	15.6 (3.9)	14.9 (3.4)	0.4555	0.6349
BIS, motor impulsivity	23.5 (4.0)	23.6 (5.8)	21.3 (3.5)	2.000	0.1381
BIS, non-planning impulsivity	23.5 (5.4)	21.7 (5.6)	24.4 (6.2)	1.6642	0.1921
ASRS, total score	8.5 (4.3)	6.6 (6.0)	7.5 (3.9)	2.1287	0.1227
Padua obsessive-compulsive, total score	13.2 (13.5)^bbb^	24.7 (25.6)^aaa^	15.4 (11.5)	6.7774	0.0014*
CHIT total score	23.1 (4.6)^bbb^	32.0 (8.7)^aaa,cc^	21.6 (2.3)^bb^	9.1285	0.0006*

BIS: Barratt Impulsivity Scale; ASRS = World Health Organization Attention-Deficit Hyperactivity Disorder Rating Scale; CHIT = Cambridge-Chicago Trait Compulsivity Scale.

Cognitive measures in the study groups are displayed in Table 3. The groups did not differ significantly on response inhibition (Stop-Signal Task, SST), nor on set-shifting (Intra-Dimensional/Extra-Dimensional Shift Task, IED). On the Cambridge Gambling Task (CGT), group differences were found on two of the three parameters (not the proportion of points gambled). The African-American group had significantly lower quality of decision-making and less risk adjustment on the CGT compared to both other groups. The Asian group did not differ significantly from the White Caucasian group on these measures.

**Table 3:** Cognitive performance in the groups. Data refer to mean (SD). Statistical tests are ANOVA. * indicates significant ANOVA, Bonferroni-corrected *p* < 0.05. Superscript letters indicate significant pairwise group differences (post-hoc *t*-tests): ^a/b/c^ p < 0.05, ^aa/bb/cc^*p* < 0.01, ^aaa/bbb/ccc^*p* < 0.001.

	Caucasian (145)^a^	African-American (*N* = 41)^b^	Asian (*N* = 16)^c^	F	P
SST, SSRT, msec	180.7 (54.1)	160.4 (44.0)	186.7 (44.6)	2.418	0.0918
IED, ED errors	8.9 (9.1)	12.0 (10.1)	10.3 (8.7)	1.5158	0.2223
CGT, proportion bet	0.54 (0.13)	0.57 (0.13)	0.51 (0.15)	1.2772	0.2812
CGT, quality of decision-making	0.96 (0.07)^bb^	0.89 (0.16)^aa,c^	0.98 (0.05)^b^	7.962	0.0005*
CGT, risk-adjustment	1.73 (1.18)^bbb^	0.38 (0.91)^aaa,cc^	1.47 (1.18)^bb^	17.880	<0.001*

IED = Intra-Dimensional/Extra-Dimensional Shift Task; SST = Stop-Signal Task; CGT = Cambridge Gamble Task. ED = Extra-Dimensional shift errors; SSRT = Stop-signal reaction times.

## 4 Discussion

To our knowledge, this is the first study to assess potential differences in impulsivity and compulsivity as a function of racial-ethnic group in recreational gamblers. The participants were young adults, included on the basis of having no mental disorders, including gambling disorder. We found important differences between groups which merit further exploration.

In terms of gambling, the African-American group endorsed significantly more subsyndromal symptoms of gambling disorder compared to the White Caucasian group. This is in keeping with epidemiological data reporting higher rates of gambling disorder in African-American people [3,8], but extends this finding to a sample of recreational gamblers without the full disorder. Interestingly, the groups did not differ in terms of the age at which they first started to gamble, so this group difference is unlikely to reflect differences in duration of gambling. The Asian group did not differ from the other groups in terms of subsyndromal symptoms of gambling disorder endorsed, which differs from a previous study among university students [10].

We did not find any significant differences between the study groups on measures of self-reported impulsivity (the Barratt Impulsivity Scale, and the ADHD rating scale), nor as indexed by the Stop-Signal Task, this latter task being a classic measure of motor disinhibition. The lack of a group difference on the Stop-Signal Task is consistent with a previous study that examined racial-ethnic differences in people with Gambling Disorder [16].

Marked differences were found between racial-ethnic groups for measures of compulsivity. Previous work in gambling disorder found higher scores on the Padua Obsessive-Compulsive inventory in black adults compared to white adults individuals [16]. Here, we found higher scores on this instrument in the African-American group compared to the White Caucasian group, again as observed in recreational gamblers. The Padua inventory is not a trans-diagnostic measure since in focuses on obsessive-compulsive symptoms and so can be considered a dimensional scale along the continuum towards OCD. By contrast, the Cambridge-Chicago Trait Compulsivity Scale (CHI-T) is trans-diagnostic [31]. This study found elevated CHI-T compulsivity in the African-American group compared to both other groups. On the neurocognitive tests, relatively worse performances on two aspects of the Cambridge Gambling Task were also found in the African-American compared to Caucasian group: risk-adjustment and quality of decision-making. Lower risk adjustment can be interpreted as a manifestation of compulsivity. Decision-making performance is particularly relevant in understanding the progression of disordered gambling because deficits on this task occur in at-risk and disordered gambling [17]. Contrary to expectation, we did not find group differences on set-shifting, which is another cognitive measure that can reflect elements of compulsivity. This may suggest that questionnaires are more sensitive to compulsivity differences between groups.

There are several limitations to the current study. The sample size was relatively small especially for the Asian group. However, it was sufficiently powered to detect clinical and cognitive differences, and there is a lack of research in this field, meaning that even small datasets may be valuable, since they can inform follow-up studies in larger cohorts. We examined a set of demographic, questionnaire, and cognitive measures that we felt may be important; as such, this is not a comprehensive evaluation of all variables that could contribute to differences in racial-ethnic group associated with recreational gambling. The current study did not include a non-gambling control group. Another limitation is that with this sample size, the maximum sampling error would be 6.9%. We feel a margin of error <10% to be reasonable for such an exploratory study. Nonetheless, follow-up studies with larger samples would be valuable. This study did not focus on other issues previously found to be relevant when considering racial-ethnic minority differences, such as social norms, acculturation, and inclusivity [[3], [4], [5], [6]]. Lastly, this study focused on young adult gamblers, and so the results may not generalize to groups with different ages.

In conclusion, this study found elevated endorsement of subsyndromal gambling symptoms, and compulsivity, as a function of racial-ethnic group in non-treatment seeking recreational gamblers. The findings may be of interest for potential early detection of transitions from recreational to disordered gambling, in the sense that they highlight domains that are already different in people who gamble recreationally but do not yet endorse diagnostic criteria for gambling disorder. These differences could constitute early treatment targets. Future studies, using a longitudinal design and variety of socio-demographic, clinical, and cognitive paradigms, may further our understanding of the influence of environmental and neurobiological factors in the development of recreational gambling, and – in turn – Gambling Disorder.
